# A Comparison between Cure Model and Recursive Partitioning: A Retrospective Cohort Study of Iranian Female with Breast Cancer

**DOI:** 10.1155/2016/9425629

**Published:** 2016-08-28

**Authors:** Mozhgan Safe, Javad Faradmal, Hossein Mahjub

**Affiliations:** ^1^Department of Biostatistics, School of Public Health, Hamadan University of Medical Sciences, Hamadan, Iran; ^2^Modeling of Noncommunicable Disease Research Center, Hamadan University of Medical Sciences, Hamadan, Iran; ^3^Research Center for Health Sciences, Hamadan University of Medical Sciences, Hamadan, Iran

## Abstract

*Background*. Breast cancer which is the most common cause of women cancer death has an increasing incidence and mortality rates in Iran. A proper modeling would correctly detect the factors' effect on breast cancer, which may be the basis of health care planning. Therefore, this study aimed to practically develop two recently introduced statistical models in order to compare them as the survival prediction tools for breast cancer patients.* Materials and Methods*. For this retrospective cohort study, the 18-year follow-up information of 539 breast cancer patients was analyzed by “Parametric Mixture Cure Model” and “Model-Based Recursive Partitioning.” Furthermore, a simulation study was carried out to compare the performance of mentioned models for different situations.* Results*. “Model-Based Recursive Partitioning” was able to present a better description of dataset and provided a fine separation of individuals with different risk levels. Additionally the results of simulation study confirmed the superiority of this recursive partitioning for nonlinear model structures.* Conclusion*. “Model-Based Recursive Partitioning” seems to be a potential instrument for processing complex mixture cure models. Therefore, applying this model is recommended for long-term survival patients.

## 1. Introduction

Breast cancer, which is the second most prevalent cancer among Iranian females [1], is the most common cause of women cancer death in the world [2]. Iran Ministry of Health has reported the age-standardized incidence rate of 33.21 per 100,000 female population [3]. Iranian patients with breast cancer are younger than the west countries patients; this faster disease formation may lead to a heavier burden [1]. Furthermore, the earlier detection of breast cancer would improve the life expectancy [4] and this is another evidence for the need of valid modeling to precisely predict the patients' hazard. A proper modeling would correctly detect the factors' effect on breast cancer, which may be the basis of health care planning [5].

Cox Proportional Hazard and Weibull Models are the two most widely used techniques to model the survival of breast cancer patients [6–9]. But admiring today's medical progressions, there is a high probability of being cured [10]. Because of this achievement, cure model is becoming more proper method especially when curability of a disease could be considered as a reality [10, 11].

The same as mixture cure model that probably allocates population individuals into one of the cured or patients groups, there are various statistical learning algorithms which divide the population into homogenous subsets. Referring to their higher accuracy and lower error rates, several articles claim the excellence of these recently introduced algorithms to their traditional counterparts [12–15]. “Model-Based Recursive Partitioning” (MoBRP) is one of the most interpretable members of this family and provides a proper power of prediction in nonlinear regression relationships [16]. This model is a hybrid tree which combines the traditional model fitting with the tree machine learning algorithm. Furthermore, MoBRP derives the benefits of regression trees such as the ability of detecting complex unknown model structures and interactions [16].

To the best of our knowledge, there is no study for modeling the survival time of Iranian breast cancer patients by using “Parametric Mixture Cure Models” (PMCM) and cautiously the only application of “Model-Based Recursive Partitioning” in survival analysis was made by Zeileis et al. to analyze German Breast Cancer dataset [17]. So the goal of this study is to compare the fitness of these two mentioned statistical methods through simulated and also practical breast cancer datasets.

## 2. Materials and Methods

### 2.1. Participants

For this retrospective cohort study, the information of 539 breast cancer patients was obtained. Approximately 37% of patients experienced death of breast cancer and the remaining were censored. These patients had been referring to Diagnostic Center of Hamedan Mahdieh Darolaytam during 1995–2013. The study entrance criteria were as follows:

- Patients who have experienced one of the lumpectomy, quadrantectomy, simple or total mastectomy, or modified radical mastectomy surgeries.
- Female breast cancer patients who underwent chemotherapy and radiotherapy before or after surgery.

The event of interest was death of breast cancer and survival time was measured in days from the date of diagnosis to the date of participants' death. Additionally, some medical prognostic and baseline characteristics factors were gathered, for example, “Human Epidermal growth factor Receptor 2” (HER2), “Progesterone Receptor Status” (PR), “Estrogen Receptor Status” (ER), and “number of involved lymph nodes.”

### 2.2. Mixture Cure Model

A basic assumption for almost all survival models is that, after sufficiently long follow-up, every individual in the population would eventually experience the event of interest. Actually this assumption is violated for some practical situations. Mixture cure is a flexible model that can overcome this limitative assumption. This model considers a subset of population as nonsusceptible. Nonsusceptible individuals are cured and would never experience the event of interest [18]. Clearly, a patient that is cured of breast cancer is nonsusceptible for experiencing the death of it.

Cured individuals would appear as censor observations during the course of follow-up. Empirical evidence for the presence of nonsusceptible individuals is the long, stable plateau which usually contains heavy censoring at the end of Kaplan-Meier survival curves [19, 20]. Provided sufficient follow-up, stabled level of probability, at the right extreme of the Kaplan-Meier, is a consistent estimator for the proportion of nonsusceptible cured patients [20].

Let *U* be the indicator variable that shows the status of being susceptible; *U* = 1 stands for susceptible or uncured patients, while *U* = 0 stands for cured individuals. Therefore, the cure model is defined as follows:

(1) $$\begin{array}{c} S\left(\;t\;|\;x,z\;\right)=\pi \left(\;z\;\right)S\left(\;t\;|\;U=1,x\;\right)+\left(\;1-\pi \left(\;z\;\right)\;\right), \end{array}$$

where *S*(*t*∣*U* = 1, *x*) is the conditional survival of susceptible individuals given the vector of covariates *x*, this probability can be modeled by one of the usual survival models such as Weibull, which is the most proper in this context [10, 11, 18, 21–25], and *S*(*t*∣*U* = 0, *x*) is the survival function of nonsusceptible individuals and is embedded as one, in the aforementioned formula.

*π*(*z*) defines the probability of being susceptible and can be modeled by one of the binary regressions such as logistic which is more common [11, 18, 23–26], as *z* is the vector of covariates and maybe the same as *x*.

Finally, *S*(*t*∣*x*, *z*) has been named marginal survival and shows the survival of the entire population.

### 2.3. Model-Based Recursive Partitioning

If a global model for all observations fits inappropriately, the total population could be split in a way that a proper fit is provided for each subset; this idea is the main motivation of MoBRP technique. This partitioning is actually a tree where each node is associated with a specific parametric model. The partitioning takes place in such a way that a stable model fitting is provided for each subset [16, 27]. More precisely, the algorithm for growing the tree is as follows:

1. Fit a parametric model to a dataset.
2. Statistically assess the stability of estimated parameters over some partitioning variables.
3. If there is an overall instability through all the estimated parameters, the population would be split along with the partitioning variable which is responsible for the most instability.
4. It should be added that splitting points are chosen in such a way that residual sum of squares or negative log-likelihood is minimized.
5. Repeat the algorithm in each terminal node.

To avoid overfitting, this kind of tree is accomplished by pre- and postpruning; prepruning is implemented via Bonferroni *p* value correction for partitioning variable selection and postpruning can be done via “Akaike Information Criterion” or “Bayesian Information Criterion” [16].

### 2.4. Simulation Study

A simulation study was planned in order to compare the performance of PMCM and MoBRP.

Data were generated from Logistic-Weibull mixture cure model [18, 28], where

(2) πz1,z2PU=1 ∣ z1,z2=exp⁡b0+b1z1+b2z21+exp⁡b0+b1z1+b2z2,St ∣ U=1,xexp⁡−exp⁡c0+c1xtρ.

In agreement with other studies [29–31], standard Normal and Uniform distributions were used for simulation. The covariates were fixed by design; *z*_1_ was generated from standard Normal distribution *z*_2_ and *x* were generated from standard Uniform distribution. The shape parameter was also fixed at *ρ* = 2.

To discover the trend of goodness of fit, the simulation was replicated 100 times at each of 36 configurations given by three levels of censoring rate, 40%, 60%, and 80% of total population; three levels of cure rate (0%, 15%, and 30%); and two levels of sample size, 500 and 1000 observations, furthermore; to survey one more complicated model structure in an additional scenario, interaction effects of *x* with *z*_1_ and *z*_2_ (i.e., *xz*_1_ and *xz*_2_) were added to the survival part of PMCM. Finally, to check the results for different shape parameters, some extra configurations were conducted for simulated samples of size 500 observations and the shape parameter of size 0.5.

### 2.5. Statistical Methods

*α*-test [20, 32] and Kaplan-Meier were used in order to check for sufficiency of follow-up and estimating the fraction of nonsusceptible individuals. Using backward variable selection, the best fit of PMCM was chosen for a Logistic-Weibull fitting. Considering the extensibility of Weibull survival distribution, it was also used for tree node modeling.

Finally, a simulation study was designed to evaluate the performance of two methods. It should be noted that “AIC” postpruning was applied to MoBRPs.

## 3. Results and Discussion

The 5-year survival rate was 68.5%. The median life time, from the time of diagnosis, was 9.02; furthermore, the population mortality rate was 36.73%. The patients' age at diagnosis was ranged from 22 to 79 and its mean (SD) and median were 46.1 (10.8) and 45 years, respectively. According to the primary information of dataset, 256 (47.49%) of individuals experienced equal or less than two involved lymph nodes and 329 (61.04%) of them the tumor size were less than two centimeters. ER^+^, PR^+^, and HER2^+^ were seen for 41.19%, 32.84%, and 76.44% of patients, respectively.

**Figure 1:**
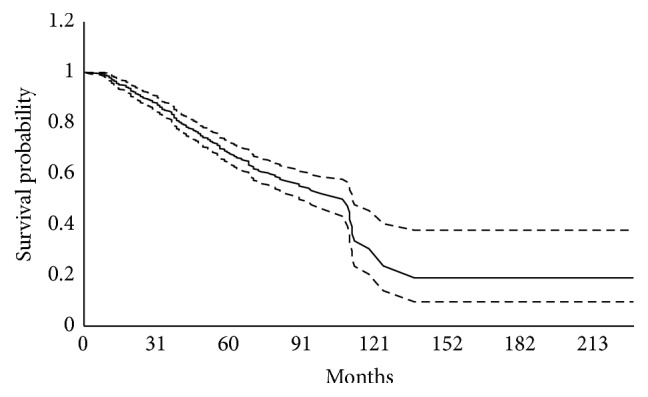
Kaplan-Meier plot of breast cancer patients' survival.

Nonparametric *α*-test rejected the insufficiency of follow-up time and as can be seen from Figure 1, Kaplan-Meier curve has been stabled at the probability of almost 0.20; this implies that 20 percent of the population is cured and nonsusceptible. The plateau tail of this curve during the study period is another visual reason of sufficient follow-up. This plot suggests “8.85 years” as the median survival time for the total population.

Table 1 shows the result of mixture cure model fitting. According to the obtained parameters for this model, the estimated mean of cure rate is about 25% of the total population. The proximity of this rate to the Kaplan-Meier estimation indicates that the crude nonparametric Kaplan-Meier method confirms its parametric counterpart in a fair manner.

The estimated parameters in the logistic part of PMCM imply that a unit increment in “tumor size” and “number of involved lymph nodes” would increase the odds of being susceptible by 1.5 and 1.1 times, respectively. The negative and positive estimated parameters, respectively, for ER^+^ and PR^+^, in the Weibull part of the cure model, also confirmed the risk and protective effects of these factors for patients with breast cancer. Based on the model, the estimated median survival time is 6.02 years for uncured and 8.03 years for the total population.

**Figure 2:**
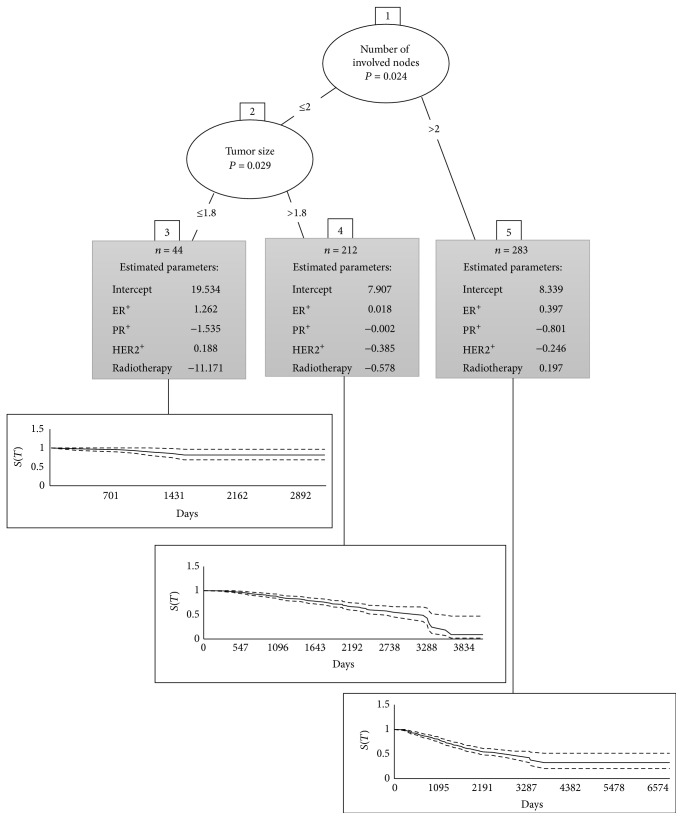
Model-Based Recursive Partitioning and Kaplan-Meier plots for each subset of population.

The fitness and estimated parameters of MoBRP are shown in Figure 2. Total population was divided according to the two partitioning variables and three terminal nodes were formed. The censoring rate for the first terminal node was 86% which was much higher than censoring rates 67% and 57%, respectively, for the second and third terminal nodes. The MoBRP resulted AIC was 3698.7, which was almost less than its counterpart of mixture cur model. This difference clarified the superior performance of MoBRP from the perspective of a full-likelihood-based criterion.

For each subset of population in terminal tree nodes, Kaplan-Meier plot was attached to the Figure 2. The most censoring rate was seen for the first terminal node where Kaplan-Meier stabled at the high probability of 0.81 and also patients in this node were associated with lower levels of the risk factors. Kaplan-Meier curve for the third terminal node decreased with a steeper slope than the plots for the first and second terminal nodes. The log-rank test reported a significant difference between the survival curves for patients belonging to the first and third terminal nodes (*p* value = 0.012). All these evidences proved that Weibull-regression-based tree could divide the population into three subsets containing low risk, high risk, and moderate risk patients; additionally low risk terminal node with heavy censoring could be considered as the node with the most cured individuals.

Tables 2 [Table tab3]–4 present the results of PMCM and MoBRP fitting for simulated data. This simulation study showed that an enhancement in cure rate would increase the “AIC”; however, smaller AICs were resulted for higher censoring rates. The same as Weibull modelling, as the number of observations decreases, “AIC” values would decrease. The comparison of simulation results, with and without interaction models, indicated that the superiority performance of PMCM or MoBRP depends on operating conditions. Smaller “AIC” is expected for PMCM, when the model structure is simple and completely known; actually this condition is so rare in medical modeling. On the other hand, as MoBRP is capable of detecting unknown covariates relationships and interactions, it would be preferred when there may exist high order of factor effects or complex structures [16]. Finally, comparison of tables with different shape parameters indicated that the mentioned trend of “AIC,” which was caused by the changes in cure and censor rates, would be the same for different shapes.

Although PMCM has been used previously to model the Iranian patients' survival [24, 25], this study is the first report to apply this technique to model the survival of Iranian women with breast cancer. Maybe the most similar studies refer to Jafari-Koshki et al. and Rahimzadeh et al. [33, 34] where a Bayesian nonmixture cure is applied to model the survival of breast cancer patients. In agreement with our investigation, the study of Jafari-Koshki et al. also determined that the effects of tumor size, number of involved nodes, and ER^+^ are to the detriment of life expectancy. Furthermore, the absence of PR expression is associated with breast cancer progression. It should be added that the harmful and beneficial effects of the above-mentioned factors are verified by PMCM in many studies related to different parts of the world other than Iran. Rondeau et al. used parametric mixture cure frailty to model the survival of breast cancer patients from the south-western of France [11]. Alike to our investigation, tumor size and number of involved nodes were the significant factors for the logistic part; the protective effect of PR^+^ was also confirmed via their survey. Forse et al. used a Weibull-Logistic PMCM and clarified the significant effect of tumor size to increase the probability of breast cancer recurrence [23]. According to their study, ER^+^ and HER2 were not effective for modeling the recurrence time among susceptible patients. Faradmal et al. have concluded similar results by means of our practical handled dataset [35]. A time-dependent Cox model was used in their analysis and maybe the application of the same dataset is the main reason for the similarity of factor effects. All these convergent results provide an almost complete guideline for clinicians in the assessing of disease progression. Obviously, prescribing an efficient treatment is conditioned on a timely accurate diagnosis and we have practically introduced PMCM to support better diagnostics.

Similar to mixture cure model which is composed of two parts, one part for attributing the probability of being susceptible and the other for modeling the survival of uncured individuals, MoBRP is a simultaneous bipartite technique of classification and survival time modeling. The only usage of MoBRP in survival analysis is referred to Zeileis et al. which applied this technique for modelling the survival of 686 German patients with breast cancer. Eight covariates were used as prognostic factors; couple of them were selected for node modeling and the remaining six factors were considered as partitioning variables. MoBRP resulted in a two-terminal-nodes tree which was formed by PR split [17].

In addition to MoBRP lower AIC, its selected split points for the partitioning variables are another marvel of its operation. Tumor size is partitioned at 1.8 centimeters which is so proximate to 2 cm as its empirical surrogate; the efficacy of this cut point is assessed by many clinical investigations [11, 23, 35–40].

## 4. Conclusions

Although MoBRP has not been designed to account for cure fraction, this survey certifies its capability to provide a fine separation of individuals with different risk levels, especially in nonlinear associations. Therefore, MoBRP seems to be a potential instrument for processing complex mixture cure models. Therefore, applying this model is recommended for long-term survival patients.

## Figures and Tables

**Table 1:** Results of Logistic-Weibull mixture cure model fitting on breast cancer patients' database.

	Estimate	Standard error	95% confidence interval		*p* value
			Lower	Upper	
Logistic part of cure model					
Intercept	−0.08	0.58	−1.22	1.05	0.89
Tumor size	0.39	0.19	0.02	0.77	0.04
Number of involved nodes	0.05	0.09	−0.11	0.22	0.53

Weibull part of cure model					
Scale	7.99	0.20	7.60	8.38	<0.05
Shape	0.62	0.04	0.54	0.70	<0.05
PR^+^	0.99	0.26	0.49	1.50	<0.05
ER^+^	−0.46	0.24	0.05	−0.93	0.05
HER2^+^	0.44	0.22	0.01	0.87	0.04
Radiotherapy	−0.43	0.21	−0.85	−0.01	0.05

AIC of cure model	3759.0				

PR^+^: being progesterone receptor positive breast cancer patient; ER^+^: being estrogen receptor positive breast cancer patient; HER2^+^: being epidermal growth factor receptor-2 positive breast cancer patient.

**Table 2:** Akaike Information Criterion (AIC) of a simulation study for a population of size 500 observations and also with the shape parameter of size 2.

	Cure rate					
	0%		15%		30%	
Model without interaction						
Censoring rate						
40%	PMCM	MoBRP	PMCM	MoBRP	PMCM	MoBRP
	751.2	787.7	862.9	864.5	1151.3	1295.7
60%	PMCM	MoBRP	PMCM	MoBRP	PMCM	MoBRP
	472.7	498.1	551.6	590.5	665.7	694.8
80%	PMCM	MoBRP	PMCM	MoBRP	PMCM	MoBRP
	233.9	245.8	272.8	288.5	315.7	330.2

Model with interaction						
Censoring rate						
40%	PMCM	MoBRP	PMCM	MoBRP	PMCM	MoBRP
	1287.9	1019.5	1384.3	1268.8	1582.7	1573.7
60%	PMCM	MoBRP	PMCM	MoBRP	PMCM	MoBRP
	822.8	747.6	841.2	739.1	907.2	823.6
80%	PMCM	MoBRP	PMCM	MoBRP	PMCM	MoBRP
	384.3	323.5	382.5	331.7	394.9	362.4

**Table 3 tab3:** Akaike Information Criterion (AIC) of a simulation study for a population of size 1000 observations and also with the shape parameter of size 2.

	Cure rate					
	0%		15%		30%	
Model without interaction						
Censoring rate						
40%	PMCM	MoBRP	PMCM	MoBRP	PMCM	MoBRP
	1397.1	1482.9	1802.1	1915.2	2256.1	2525.1
60%	PMCM	MoBRP	PMCM	MoBRP	PMCM	MoBRP
	926.9	975.8	1106.6	1181.6	1312.6	1309.9
80%	PMCM	MoBRP	PMCM	MoBRP	PMCM	MoBRP
	489.3	519.3	550.6	572.7	613.6	650.6

Model with interaction						
Censoring rate						
40%	PMCM	MoBRP	PMCM	MoBRP	PMCM	MoBRP
	2583.8	2209.8	2793.4	2442.1	3180.7	3032.7
60%	PMCM	MoBRP	PMCM	MoBRP	PMCM	MoBRP
	1621.3	1492.2	1683.7	1416.0	1813.7	1525.5
80%	PMCM	MoBRP	PMCM	MoBRP	PMCM	MoBRP
	730.0	625.0	758.9	635.1	782.9	693.4

**Table 4:** Akaike Information Criterion (AIC) of a simulation study for a population of size 500 observations and also with the shape parameter of size 0.5.

	Cure rate					
	0%		15%		30%	
Model without interaction						
Censoring rate						
40%	PMCM	MoBRP	PMCM	MoBRP	PMCM	MoBRP
	1856.18	1861.86	2494.85	2546.09	2729.86	2778.33
60%	PMCM	MoBRP	PMCM	MoBRP	PMCM	MoBRP
	1066.41	1080.39	1641.47	1638.89	1646.18	1707.90
80%	PMCM	MoBRP	PMCM	MoBRP	PMCM	MoBRP
	457.14	477.88	814.69	851.95	848.36	874.43

Model with interaction						
Censoring rate						
40%	PMCM	MoBRP	PMCM	MoBRP	PMCM	MoBRP
	3090.44	2751.21	3153.18	2911.67	3458.61	3220.91
60%	PMCM	MoBRP	PMCM	MoBRP	PMCM	MoBRP
	1801.99	1641.78	1825.50	1674.54	1989.8	1869.79
80%	PMCM	MoBRP	PMCM	MoBRP	PMCM	MoBRP
	752.21	652.69	770.28	654.37	772.32	672.06
